# Negative modulation of mitochondrial calcium uniporter complex protects neurons against ferroptosis

**DOI:** 10.1038/s41419-023-06290-1

**Published:** 2023-11-25

**Authors:** Alejandro Marmolejo-Garza, Inge E. Krabbendam, Minh Danh Anh Luu, Famke Brouwer, Marina Trombetta-Lima, Osman Unal, Shane J. O’Connor, Naďa Majerníková, Carolina R. S. Elzinga, Cristina Mammucari, Martina Schmidt, Muniswamy Madesh, Erik Boddeke, Amalia M. Dolga

**Affiliations:** 1 Faculty of Science and Engineering, Department of Molecular Pharmacology, Groningen Research Institute of Pharmacy (GRIP), University of Groningen, 9713 AV Groningen, The Netherlands; 2 Department of Biomedical Sciences of Cells & Systems, Section Molecular Neurobiology, Faculty of Medical Sciences, University of Groningen, University Medical Center Groningen, Groningen, The Netherlands; 3 Department of Pathology and Medical Biology, University Medical Center Groningen, University of Groningen, Groningen, The Netherlands; 4 Department of Biomedical Sciences, University of Padua, 35131 Padua, Italy; 5 Department of Medicine/Cardiology, Center for Mitochondrial Medicine, University of Texas Health San Antonio, San Antonio, TX 78229 USA

**Keywords:** Apoptosis, Cell death in the nervous system

## Abstract

Ferroptosis is an iron- and reactive oxygen species (ROS)-dependent form of regulated cell death, that has been implicated in Alzheimer’s disease and Parkinson’s disease. Inhibition of cystine/glutamate antiporter could lead to mitochondrial fragmentation, mitochondrial calcium ([Ca^2+^]_m_) overload, increased mitochondrial ROS production, disruption of the mitochondrial membrane potential (ΔΨ_m_), and ferroptotic cell death. The observation that mitochondrial dysfunction is a characteristic of ferroptosis makes preservation of mitochondrial function a potential therapeutic option for diseases associated with ferroptotic cell death. Mitochondrial calcium levels are controlled via the mitochondrial calcium uniporter (MCU), the main entry point of Ca^2+^ into the mitochondrial matrix. Therefore, we have hypothesized that negative modulation of MCU complex may confer protection against ferroptosis. Here we evaluated whether the known negative modulators of MCU complex, ruthenium red (RR), its derivative Ru265, mitoxantrone (MX), and MCU-i4 can prevent mitochondrial dysfunction and ferroptotic cell death. These compounds mediated protection in HT22 cells, in human dopaminergic neurons and mouse primary cortical neurons against ferroptotic cell death. Depletion of MICU1, a [Ca^2+^]_m_ gatekeeper, demonstrated that MICU is protective against ferroptosis. Taken together, our results reveal that negative modulation of MCU complex represents a therapeutic option to prevent degenerative conditions, in which ferroptosis is central to the progression of these pathologies.

## Introduction

Recently, a form of cell death named ferroptosis was discovered and characterized in a multitude of neurodegenerative diseases [1–4]. Ferroptosis is an iron-dependent oxidative stress-driven [5] cell death [6–11]. Hallmarks of ferroptosis are related to alterations in mitochondrial morphology and function [12], including mitochondrial fragmentation, mitochondrial calcium ([Ca^2+^]_m_) overload, increased mitochondrial reactive oxygen species (ROS) production [13], lipid peroxidation and disruption of the mitochondrial membrane potential (ΔΨ_m_) [14]. Mitochondrial dysfunction hallmarks such as these occur in neurodegeneration [15, 16]. Ferroptosis is also associated with dysregulated cytosolic Ca^2+^ levels [17–19]. Induction of the multi-step ferroptotic cell death cascade can be initiated by glutamate and small molecules, erastin or RSL-3. Erastin or extracellular application of glutamate inhibit the glutamate/cystine antiporter system, the most upstream player in ferroptosis pathway [5]. Inhibition or blocking of the glutamate/cystine antiporter results in a decreased glutathione (GSH) synthesis [20], as cysteine is the rate-limiting factor in GSH synthesis. Reduction in GSH levels diminishes the antioxidant response of cells, thereby increasing ROS levels which induces caspase-independent cell death [21, 22]. GSH depletion impairs the activity of glutathione peroxidase-4 (GPX4, target of RSL-3) and increases 12/15-lipoxygenase (LOX12/15) function, both of which are crucial steps upstream of mitochondrial dysfunction [23].

Pedrera et al. demonstrated that cytosolic calcium increases over time with the ferroptotic inducers erastin and RSL3 and this is prevented by ferroptosis inhibitors [18]. However, mitochondrial calcium overload was not studied. We have shown that ferroptosis/oxytosis process is associated with the reduction of mitochondrial respiration and [Ca^2+^]_m_ overload [14, 19, 24]. These hallmarks were reverted with a concomitant restoration of cell viability with small conductance Ca^2+^-activated K^+^ (SK) channel activation. Furthermore, we have modeled a condition of increased ER-mitochondria tethering where there is increased basal [Ca^2+^]_m,_ with an increased oxidative stress and sensitivity to ferroptosis. SK channel activation confers protection in these conditions of oxidative stress by decreasing [Ca^2+^]_m_ [25]. With these antecedents, it is logical to think of [Ca^2+^]_m_ uptake and modulators of mitochondrial calcium uniporter (MCU) complex, the main entry of Ca^2+^ into mitochondria, as a potential target to prevent ferroptosis. Protective effects of inhibition of [Ca^2+^]_m_ uptake have been reported, such as treatment with Nelfinavir or Ruthenium Red (RR) against HT22 cells exposed to oxygen-glucose deprivation in vitro and in an ischemia/reperfusion in vivo model [26].

The MCU was identified as a highly selective channel that mediates Ca^2+^ influx into the mitochondria [27–29]. The MCU complex contains regulatory elements in the form of a heterodimer (MICU1/MICU2) that prevent Ca^2+^ overload. Continuous [Ca^2+^]_m_ influx at resting membrane potential due to the electrogenic driving force can lead to cell damage [30, 31]. The effect of MCU in mitochondrial Ca^2+^ homeostasis makes it a potential target against ferroptotic cell death.

Antagonism of the MCU by the organic staining dye, ruthenium red (RR), has been shown to regulate Ca^2+^ levels and mitigate ROS damage in vitro in oxytosis-mediated cell death [32]. The FDA approved compound mitoxantrone (MX) was identified as an MCU inhibitor in an orthogonal screening of yeast mitochondria reconstituted with human MCU and aequorin [33]. MX docks into the MCU pore with high specificity, decreasing [Ca^2+^]_m_ in a dose-dependent manner, without influencing the function of other ion channels. The fact that MX has already received regulatory approval makes the drug an exciting candidate to treat diseases with [Ca^2+^]_m_ overload [34]. Authors then validated the effects of MX on [Ca^2+^]_m_ in mammalian cell assays [33]. MX is a well-known chemotherapeutic drug. As suggested by Arduino et al., it is possible that the anti-neoplasic and MCU-inhibitory activities derive from different structural moieties within MX. Ru265 is a cell-permeable modified ruthenium-containing compound with increased specificity and neuroprotective potential against hypoxic/ischemic (HI) brain injury [35]. The anti-ferroptotic effects of Ru265 have not yet been assessed. The high-throughput screening (HTS) from Di Marco et al. proposed MCU-i4 and MCU-i11 to have attenuating effect on [Ca^2+^]_m_ uptake in vitro and ex vivo MICU1 binding. All above mentioned compounds which attenuate [Ca^2+^]_m_ may prove beneficial to modulate ferroptosis.

In this study, we aim to investigate the effects of negative modulation of the MCU on ferroptotic cell death. We profiled the effects of RR, its derivative Ru265, MX as well as the MICU1 binding compounds MCU-i4 and MCU-i11 on ferroptotic cell death, ferroptosis hallmarks, and [Ca^2+^]_m_. We also investigated whether a decrease in the expression of the mitochondrial calcium uptake gatekeeper protein MICU1 potentiates cell death during ferroptosis via mitochondrial calcium overload.

## Methods

### Cell culture and compounds

HT22 cells were cultured in plastic culture flasks in Dulbecco’s Modified Eagle Medium (DMEM; Gibco, Thermo Fisher Scientific) supplemented with 10% fetal bovine serum (FBS; GE Healthcare Life Sciences), 100 U/mL penicillin, 100 µg/mL streptomycin (Gibco, Thermo Fisher Scientific), and 1 mM sodium pyruvate (Gibco, Thermo Fisher Scientific) at 37 °C and 5% CO_2_. For siRNA transfection experiments, penicillin and streptomycin were omitted from the culture medium to mitigate interference with the transfection. Cells were treated with various compounds; erastin (Tocris bioscience), ruthenium red (RR; Abcam), mitoxantrone dihydrochloride (MX; Sigma-Aldrich), ferrostatin (Fer; Sigma-Aldrich), cell-permeable N-cyclohexyl-N-[2-(3,5-dimethyl pyrazole-1-yl)-6-methyl-4-pyrimidinamine (CyPPA, provided by Prof. Dr. F.J. Dekker, University of Groningen), Ru265 (provided by Joshua Woods and Justin Wilson, Cornell University), DS16570511 (Cayman chemical). Ferroptosis was initiated by glutamate, erastin or RSL-3. In HT22 cells, glutamate induces ferroptosis in a similar way as erastin by inhibiting glutamate/cystine transporter resulting in oxidative stress. Due to the absence of NMDA receptors [36–38] in HT22 cells, glutamate is not able to mediate excitotoxicity.

Mouse Embryonic Fibroblasts (MEF; Kindly provided by Cristina Mammucari) were cultured in Dulbecco’s Modified Eagle’s Medium (DMEM; Gibco) supplemented with 10% Fetal Bovine Serum (FBS; Gibco), 1% penicillin and streptomycin (p/s; Gibco) and 1 mM sodium pyruvate (Gibco) at 37 °C in a humidified incubator with 5% CO_2_. Cultures were passed when confluent. During passing, cells were washed with 1x PBS and detached with 2 ml 1x Trypsin-EDTA (Biowest; L0930-100). After detaching, 10 ml culture media was added, and cells were centrifuged for 5 min at 300 rcf. Pellet was resuspended in 5 ml culture media and counted with a counting chamber. Cells were seeded with a density of 400,000 cells on Mondays and Wednesday and 300,000 cells on Fridays, in T75 flask. 300,000 cells/well in 6-well plates for RNA extraction or Western Blot were seeded overnight. Cells were treated with following compounds: RSL3 (Selleckchem; s8155), PD146176, Ferrostatin-1 (Sigma- Aldrich; SML0583) and MCU-i4 (Tocris; 7195).

Postmitotic differentiated human dopaminergic neuronal cells LUHMES were used in these experiments. Undifferentiated LUHMES cells were proliferated in cell culture flasks (Nunclon DELTA surface) coated with 0.1 mg/ml poly-L-Lysine (PLL). For experiments, cell culture dishes were coated with 0.1 mg/ml PLL overnight and washed three times with sterile water, followed by coating with 5 µg/ml fibronectin (Sigma-Aldrich, St. Louis MO, USA) overnight in the incubator (37 °C, 5% CO_2_). Before plating the cells, fibronectin was removed, and the wells were washed with phosphate-buffered saline (PBS) and dried. Cells were plated at a density of 55,000/cm^2^ in Dulbecco’s modified Eagle’s medium (DMEM)/F12 (Sigma-Aldrich, St. Louis MO, USA) with 1% N2-supplement (Life Technologies, Carlsbad, CA, USA), 0.04 µg/ml basic fibroblast growth factor (R&D Systems, Minneapolis, MN, USA). After 24 h of plating, the medium was exchanged to differentiation medium DMEM/F12 with 1% N2-supplement, 1 µg/ml tetracycline, 0.49 mg/ml dibutyryl cyclic AMP (Sigma-Aldrich, St. Louis MO, USA) and 2 ng/ml glial cell-derived neurotrophic factor (R&D Systems, Minneapolis, MN, USA). All cell lines were tested regularly for mycoplasma contamination.

### Primary cortical neuron preparations

Primary cortical neurons (PCN) were prepared from C57BL/6 embryonic (E13-14) mice (mixed gender) under sterile conditions [39]. The cortex from embryos were collected and treated with 0.2 mg/ml trypsin at 37 °C for 15 min. After DNase and trypsin inhibitor treatment, the cell pellet was obtained through centrifugation. Neurobasal medium (#2508186; Gibco, Thermo Fisher Scientific, Netherlands) supplemented with 100 U/ml penicillin, 100 μg/ml streptomycin (#15070–063; Gibco, Thermo Fisher Scientific, Netherlands), 2 mM l-glutamine (#15070063; Gibco, Thermo Fisher Scientific, Netherlands) and 2% B27 supplement (#17504/044; Gibco, Thermo Fisher Scientific, Netherlands) was used to resuspend the cell pellet, followed by being seeded on PEI-coated (polyethylenimine, #P3143) 12-well Ibidi plates (30,000 cells/well). Experiments were performed on day in vitro (DIV) 7–10. Each experiment was independently repeated 3 times.

### Cell viability measurements

Quantification of cell viability was achieved using a 3-(4,5-dimethylthiazol-2-yl)-2,5-diphenyltetrazolium bromide (MTT; Sigma-Aldrich) colorimetric reduction assay. Following incubation, MTT (0.5 mg/ml) was removed from the wells and then incubated again at −20 °C for at least 1 h. The purple formazan was dissolved using DMSO and incubated (120 rpm) at 37 °C for 30 min. The absorbance was quantified using a Synergy H1 Multi-mode reader (Biotek, Winooski, VT, USA) at 570 nm with a reference filter of 630 nm. Control conditions were set to represent 100% cell viability. *N* = 6 technical replicates every experiment. Every experiment was replicated at least 3 times with similar results. Alternatively, real-time cell impedance was used as a measure of cell viability using xCELLigence (Agilent Biosciences, San Diego, CA). Changes in impedance were measured using the Real-Time Cell Analyzer RTCA-MP (Roche, Germany). Cell index values were normalized to 1 just before the application of compounds, which is designated as the starting point (t = 0 h) of the experiment. *N* = 6 technical replicates every experiment. Every experiment was replicated at least 3 times with similar results.

The quantification of apoptosis and necrosis was carried out using the fluorescent dyes AnnexinV-FITC and propidium iodide (PI) (Invitrogen, Thermo Fisher Scientific, Carlsbad, CA, USA), respectively. Cells were incubated in binding buffer containing the dyes at room temperature (RT) for 5 min. Flow cytometric analysis was performed with excitation at 488 nm and emission at 525/530 nm for AnnexinV (FITC filter), and at 690/50 nm for PI (PE filter), using the CytoFLEX (Beckman Coulter, Woerden, the Netherlands). AnnexinV-, PI- and AnnexinV^+^PI^-^ positive cells were counted as dead cells. Data were collected from 15 000 cells in triplicate per condition. Every experiment was repeated 3 times, and a representative experiment is shown in the figures.

### Measurement of mitochondrial calcium

Quantification of mitochondrial calcium was carried out using rhodamine-2-acetoxymethyl ester dye (Rhod2-AM; Life technologies). Rhod2-2AM co-localizes with mitochondria (Fig. S3D). Cells were incubated with 2 µM Rhod2-AM in DMEM without serum or antibiotics for 30 min at RT in the dark, followed by incubation of the cells in DMEM containing serum and antibiotics for 30 min in the dark. After incubation and collection in PBS, fluorescence was excited at 552 nm and recorded at 581 nm using the CytoFLEX. Data were collected from 15,000 cells in triplicate per condition. Unless stated otherwise, every experiment was repeated 3 times and a representative experiment is shown in the figures.

### Measurement of lipid peroxidation

The level of lipid peroxidation within the cell was assessed using the fluorescent dye boron-dipyrromethene (BODIPY; Fisher Scientific, Landsmeer, Netherlands). Cells were incubated with 2 µM BODIPY at 37 °C for 1 h. Following incubation, cells were collected in PBS. Fluorescence was excited 488 nm and detected at 530 nm and 585 nm using the CytoFLEX. Data were collected from 15,000 cells in triplicate per condition. Every experiment was repeated 3 times and a representative experiment is shown in the figures.

### Measurement of mitochondrial membrane potential

Loss of the mitochondrial membrane potential was determined with tetramethylrhodamine-ethyl ester (TMRE; Fisher Scientific, Landsmeer, Netherlands) dye. Cells were collected and incubated 30 min with 0.2 µM TMRE at 37 °C. TMRE fluorescence was excited at 488 nm and detected at 690/50 nm using CytoFLEX. Data were collected from 15,000 cells in triplicate per condition. Every experiment was repeated 3 times and a representative experiment is shown in the figures.

### Quantitative real-time PCR

RNA was isolated using TRIZOL reagent (TRI Reagent Solution, Applied Biosystems, the Netherlands) according to manufacturer’s protocol. Total RNA yield was measured using the NanoDrop 1000Spectrometer (Thermo Fisher Scientific, Wilmington, DE, USA). Equal amounts of cDNA were synthesized using Reverse Transcription System (Promega, Madison, WI, USA), according to manufacturer’s protocol. For qPCR, cDNA samples were mixed with SYBR green, forward primers, and reverse primers (see Supplementary Table 1). The qPCR was run on an Eco Real-Time PCR System (Illumina, CA, USA) and the protocol was as follows: 30 min polymerase activation at 95 °C. PCR cycling divided into 3 stages, denaturation for 30 min at 95 °C, annealing for 30 min at 54 °C and elongation for 30 min at 72 °C. The total cycle count was 45 cycles. Incubation lasted for 5 min at 72 °C. Finally, the melt curve was generated using the following protocol; 15 s at 95 °C, 15 s at 55 °C and a final 15 s at 95 °C. qPCR data were analyzed on LinRegPCR software.

Cells were washed with 1x PBS and lysed in lysis buffer RA1 (Macherey- Nagel GmbH & co; Lot 95018) supplemented with 10% DTT. Cells were collected by scraping and stored at −20 °C till further use. RNA was isolated using RNA purification kit (Macherey-nagel; 740966.50). cDNA was synthesized with 0,5 µg random hexamer primers per µg total RNA. 1 µl 10 mM dNTP Mix (U151A; Promega) was added and heated in a Thermal Cycler (VWR) for 5 min to 70 °C. 100 units M-MLV rev. Transcriptase (A356A; Promega), 20 units RNasin Ribonuclease inhibitor (N251B; Promega;) and 1x M-MLV Reaction buffer (M531A; Promega) were added and cDNA synthesis was performed at 25 °C for 10 min, 37 °C for 50 min and 70 °C for 15 min. The cDNA was diluted to a final concentration of 10 ng/µl in RNAse-free water. qPCR was performed in the presence of FastStart Universal SYBR Green Master (06 402 712 001; Roche) using QuantStudio(TM) 7 Flex System (278870976). PCR started with 10 min at 95 °C for polymerase activation. PCR cycling was: 95 °C for 30 s, annealing at 55 °C for 30 s, and extension at 72 °C for 30 s, for 45 cycles with a final melt curve: 5 min incubation at 72 °C, 15 s at 95 °C, 15 s at 55 °C and 15 s at 95 °C. Geometric mean of the reference genes RPL3A, GAPDH and HPR1 was used for normalization. Mouse mRNA primers used were purchased from Biolegio and the sequences are listed in Appendix 1a. *N* = 3 different passages for qPCR experiments.

### Seahorse XF analysis

HT22 cells were seeded in Seahorse XF 96-well plates (Seahorse Biosystems, Agilent Technologies, Waldbronn, Germany) and treated with erastin, MX, and RR. On the day of the measurement, medium was removed and replaced with assay buffer, consisting of base medium (0.8 mM MgSO4, 1.8 mM CaCl_2_, 143 mM NaCl, 5.4 mM KCl, 0.91 mM NaH_2_PO4, 3 mg/L Phenol Red) 1 mM sodium pyruvate, 2 mM L-Alanyl-L-Glutamine and 25 mM D-Glucose (pH 7.35) and incubated at 37 °C (without CO_2_) for 1 h. Seahorse XF was used to measure oxygen consumption rate (OCR) and extracellular acidification rate (ECAR). Three baseline measurements (3x min mix, 0-min delay, 3-min measure = 3/0/3) were recorded. Mitochondrial metabolism was assessed by injection of 4 µM oligomycin (3/0/3), 50 µM dinitrophenol (DNP), 0.1 µM Rotenone/1 µM Antimycin A (3/0/3), and 50 mM 2-deoxy-D-glucose (2-DG) (3/0/3). OCR and ECAR were determined after injection of each compound. Immediately after analysis, protein content was quantified using Pierce BCA Protein Assay Kit (Fisher Scientific). These data were then used to normalize data obtained in the Seahorse analysis. *N* = 6 measurements per condition. Each experiment was repeated 3 times with similar results and a representative experiment is shown.

### siRNA transfection

HT22 cells were transfected with 100 nM siRNA to knock down the MICU1 regulatory hand of the MCU. 25 nM scrambled siRNA was used as a negative control, with transfection reagent alone used as a regular control. To prevent interference of antibiotics with siRNA delivery and gene silencing, the cells were plated using antibiotic-free medium. ON-TARGETplus MICU1 siRNA (Dharmacon, Cambridge, U.K) and DharmaFECT 2 transfection reagent (Dharmacon, Cambridge, U.K) were prepared in two separate tubes of an equal volume containing serum-free medium and incubated for 5 min at RT. Following incubation, the tubes were combined and incubated for a further 20 min at RT. Serum-free medium was added to the combined tube to reach the desired working concentration of siRNA. The appropriate volume of transfection medium replaced the medium in the well plates. Cells were incubated at 37 °C and 5% CO_2_ for 24–48 h. *N* = 6 technical replicates per condition. Each experiment was repeated 3 times with similar results and a representative experiment is shown.

### Western blotting

Cells were washed with 1x PBS and lysed in RIPA buffer, containing 125 mM NaCL (1.06404.1000; EMSURE), 25 mM Tris-Cl (pH 7.4) 1% Triton X-100 (X100-500M; Sigma-Aldrich), 0.5% sodium deoxycholate (D6750-500G; Sigma-Aldrich), 0.1% SDS (L5750-1KG; Sigma-Aldrich) and 1x complete EDTA-free protease inhibitors. Cells were collected by scraping and centrifuged at 4 °C for 15 min at max speed. Supernatant was transferred to a new tube and stored at −20 °C till further use. Pierce^TM^ BCA protein assay kit (Thermo Scientific) was used to determine the protein concentration of the sample. The samples were adjusted with Ultra-Pure (UP) water and SDS loading buffer to obtain 0.65–1 ug/µl protein per condition. The samples were run on 10% SDS-PAGE and blotted to a methanol activated Roti®-PVDF blotting membrane (ROTH). The primary antibodies, rabbit anti-CBARA1 (1:250, Novusbio) and rabbit anti- vinculin (1:10,000, Sigma-Aldrich) were detected with HRP-conjugated antibodies. Antibodies were diluted in 5% milk in TBST solution. The bands were detected using Western Lightning Plus-ECL (PerkinElmer) in G-Box (SYNGENE). Acquired photos are in the Supplementary Material. The housekeeping gene Vinculin was used as a loading control. *N* = Different passage per collection.

### Mitochondria morphological quantification

Cells were seeded in a 24-well plate and treated the following day. Medium was replaced 16 h post-treatment with medium containing 200 nM mitotracker deep red (Thermo Fisher Scientific) in dark conditions. Next, the plate was incubated at 37 °C and 5% CO_2_ for 30 min. Following incubation, cells were ready for fixated with 4% PFA and incubated at RT for 25 min. Samples were mounted onto coverslips using fluoroshield (Sigma-Aldrich). Upon imaging, mitochondria were categorized from I-IV. Category I displayed elongated mitochondria that were distributed throughout the cell. Category II mitochondria were elongated and partially fragmented. Category III mitochondria were fragmented and accumulated around the nucleus. Category IV mitochondria exhibited further fragmentation and rounding of the cells. Mitochondria morphology quantification was carried out using a Leica DM4000B fluorescent microscope (Leica, Amsterdam, the Netherlands). Images were captured using the TXR filter at ×40 magnification and then processed using Las v4.3 software (Leica).

### HT22 MICU1 KO generation

KO were generated with the CRISPR/CAS9 system. Oligos were designed on the second exon junction, on CDS starting point, by crispor.tefor.net. Oligos were annealed and 5’ phosphorylated in the presence of T4 polynucleotide kinase (PNK), for 30 min at 37 °C, 95 °C for 5 min with a final rampdown to 20 °C at a rate of 5 °C per minute. 200x diluted guides were inserted in PsPCas9(BB)-2A-puro PX459 vector (62988; Addgene) with 1x Tango buffer (Thermo Scientific; BY5), 10 mM Dithiothreitol (DTT), 10 mM ATP (Biolabs new england; P0756S), BbsI (Thermo Scientific; ER1011) and T4 polykinase (Promega; M410A) in T100TM Thermal Cycler (Bio-rad) with following program: 6 cycles of 37 °C for 5 min and 21 °C for 5 min, followed by 37 °C for 7 min. 25 µL of competent bacteria were transformed with 5 µL of the ligation reaction and incubated for 10 min on ice. Cells were heat shocked for 45 s at 42 °C and placed on ice or 2 min. Bacteria were recovered in 500 µL SOC medium at 37 °C, 240 rpm for 1 h. After recovery, 100 µL of the product was distributed by beads on LB agar plates containing 100 µg/ml ampicillin and incubated at 37 °C, 240 rpm for 16 h. Colonies were picked and grown in 100 µl LB, containing 100 µg/ml ampicillin in 96-well plate for 16 h. Positive insertions were selected using PCR and gel electrophoresis. Positive colonies were grown for 16 h at 37 °C, 240 rpm in 5 ml LB medium. Plasmids were obtained using PureLinkeTM Quick Plasmid Miniprep Kit (Invitrogen; K210010) and sequenced.

### Calcein measurements of neurite area in LUHMES cells and primary cortical neurons (PCNs)

On differentiation day 7, LUHMES cells were treated for 6 h with RSL3 and MCUi4. On in vitro day 7, PCNs were treated for 18 h with RSL3 and MCUi4. Neuronal network integrity was assessed with calcein-AM 1 uM and Hoechst 1 ug/uL (1 h incubation at 37 degrees in CO2 incubator). After 1-h, confocal microscopy images were acquired using Cell Discoverer 7 (Zeiss). A Z-stack was acquired, and a Maximum Intensity Projection was generated. Live cells are distinguished by the ubiquitous intracellular esterase activity, determined by the conversion of the cell-permeant calcein AM to the intensely fluorescent calcein. The nuclei area (soma) was subtracted from the green area (neurites and soma). Green area was corrected by the number of nuclei quantified to obtain the readout of neurite area (µm^2^/cell) per picture/field. *N* = 10–20 ROI per condition. Every experiment was repeated 3 times with similar results. A pooling of all measurements across experiments is shown.

### Statistical analysis

All data displayed are mean ± SEM unless otherwise indicated in the figure legend. Statistical analyses were carried out using Prism 8 (GraphPad Software, San Diego, USA) and statistical comparison was performed using one-way ANOVA followed by Tukey’s test for multiple comparisons for experiments where there was only one column factor. For experiments that investigated co-treatments and had two factors, statistical comparison was performed using two-way ANOVA. Experiments were repeated at least three independent times with cells of different passage numbers and with 3–6 technical replicates per condition. For primary cortical neuron experiments, the experiment was repeated with 3 different animals. *p* values illustrating statistically significant differences between the mean values are defined as *p < 0.05, **p < 0.01, ***p < 0.001.

## Results

### Ruthenium red prevents ferroptotic cell death

To study ferroptosis, we used the HT22 murine hippocampal cell line, an established model for ferroptosis and oxytosis [14]. We tested the effects of ferroptosis-inducing agents erastin, RSL3, and glutamate on cell viability. Bright-field microscopy revealed a decrease in cell confluency, alongside with cell rounding (Supplementary Fig. 1A), a morphological characteristic of cell death [40–42]. We employed the known ferroptosis inhibitors, ferrostatin and PD-146176 to prevent cell death. PD-146176 acts as a LOX inhibitor [43], thereby attenuating lipid peroxidation in the cell [44, 45] and ferrostatin, a commonly used ferroptotic inhibitor which is a free-radical trapping anti-oxidant [46] and prevents damage to membrane lipids. PD-146176 (Supplementary Fig. 1B, C) and ferrostatin (Supplementary Fig. 1D) prevented erastin-induced ferroptosis. These inhibitors also prevented glutamate-induced cell death (Supplementary Fig. 1E, F) and RSL3-induced cell death (Supplementary Fig. 1G) in HT22 cells.

**Fig. 1:**
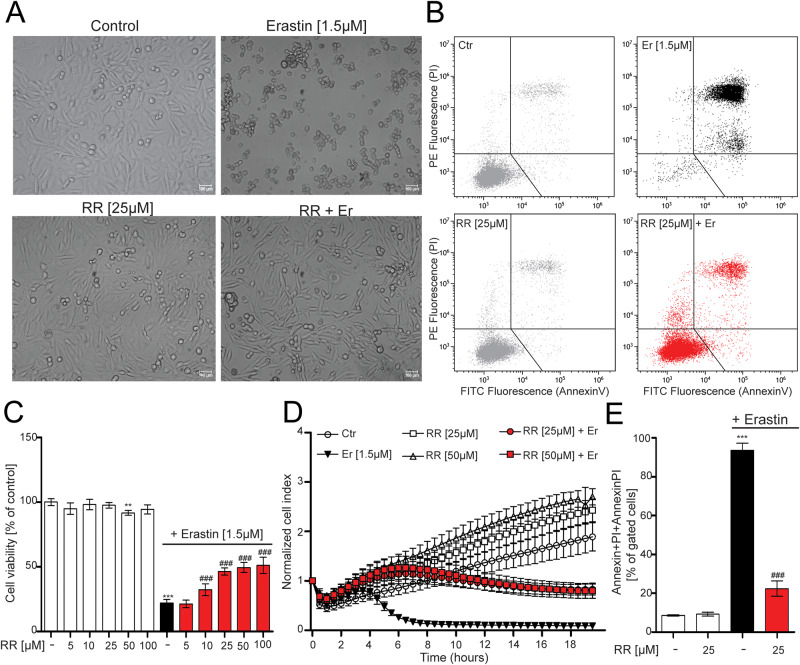
Attenuating mitochondrial calcium uptake with ruthenium red is protective against ferroptosis. **A** Microscope images of cells treated with ruthenium red (RR) (25 µM) and co-treatment of erastin (1.5 µM). Scale bar = 100 µm. **C** MTT measurement showing cell viability after RR (5–100 µM) and erastin (1.5 µM) treatment. **D** xCELLigence measurement of real-time cell impedance following RR treatment (25 µM–50 µM) in the presence or absence of erastin (1.5 µM). **E** Annexin/PI staining upon administration of RR alone (25 µM) or co-administered with erastin, representative panels shown in (**B**). Data are presented as mean ± SEM. Two-way ANOVA with Dunnett’s multiple comparisons test. ***p < 0.001, compared to untreated control, ^##^*p* < 0.01, ^###^*p* < 0.001 compared to erastin alone.

Next, we evaluated the protective potential of pharmacological modulators of the MCU complex against erastin-induced ferroptosis. We started with RR, as one of the most studied pharmacological inhibitor of MCU, and observed that co-treatment with RR prevented erastin-induced cell death morphological changes (Fig. 1A), and viability loss (Fig. 1C). RR prevented the erastin-induced reduction in cell viability in a concentration-dependent manner with a plateau at 25 µM. We then monitored in real-time the cell impedance by xCELLigence real-time impedance measurements [47]. Changes in cell impedance, (depicted as the cell index) reflect alterations in cell morphology. Erastin decreased the cell index in 10 h, as the cell morphology was drastically reduced, while healthy cells continued to increase the cell index, due to cell proliferation. RR alone showed similar cell index values as the healthy cells, while in the combination with erastin prevented the decrease of the cell index of erastin-treated cells (Fig. 1D). In addition, RR co-treatment decreased the frequency of double positive cells in Annexin/PI FACS analysis (Fig. 1B, E). Taken together, these results suggest that RR is effective in attenuating erastin-induced neuronal cell death.

### MCU antagonism using mitoxantrone is protective against ferroptosis

**Fig. 2:**
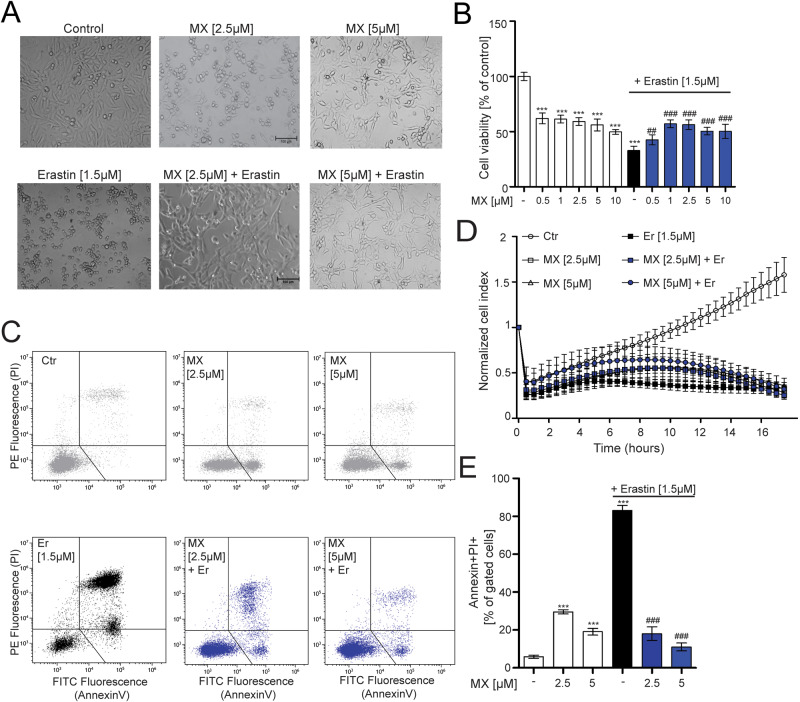
Antagonism of MCU using mitoxantrone is protective against ferroptosis. **A** Microscope images of cells treated with MX (2.5–5 µM) and co-treatment of erastin (1.5 µM). Scale bar = 100 µm. **C** MTT measurement showing cell viability after MX (0.5–10 µM) and erastin (1.5 µM) treatment. **D** xCELLigence measurement of real-time cell impedance following MX treatment (2.5–5 µM) in the presence or absence of erastin (1.5 µM). **E** Annexin/PI (early apoptosis and late apoptosis/necrosis) staining upon administration of MX alone (2.5–5 µM) or co-administered with erastin, representative panels shown in (**B**). Data are presented as mean ± SEM. Two-way ANOVA with Dunnett’s multiple comparisons test. ***p < 0.001, compared to untreated control, ^##^*p* < 0.01, ^###^*p* < 0.001 compared to erastin alone.

We next investigated MCU antagonism with MX [33] on erastin-induced ferroptosis. Co-treatment of MX with erastin appeared to restore cell morphology and cell confluency in comparison to erastin-treated cells (Fig. 2A). However, we observed a reduction in cell survival and an alteration of cell morphology compared to control when MX was administered alone, as detected by MTT assay, and xCELLigence measurements (Fig. 2B, D). Although Annexin/PI analysis showed a slight increase in early apoptotic cells treated with MX alone (Fig. 2C, E), co-treatment of MX and erastin improved cell viability when compared to erastin alone (Fig. 2B–E). Co-treatment with MX attenuated erastin-induced cell death.

### RR and MX reduce hallmarks of ferroptosis

Erastin has already been shown to increase [Ca^2+^]_m_, mitochondrial ROS, lipid peroxidation, and disruption of the ΔΨ_m_, hallmarks of ferroptosis [48–51]. We next investigated whether inhibition of mitochondrial [Ca^2+^] uptake by MX or RR can prevent erastin-mediated ferroptosis pathway. To this end, we used the fluorescent dyes Bodipy, MitoSOX and TMRE to measure changes in lipid peroxidation, mitochondrial ROS and ΔΨ_m_ loss, respectively. Particularly, we were interested whether [Ca^2+^]_m_ is changed by these compounds. We observed a decrease in [Ca^2+^]_m_ when RR and MX were applied alone a concentration-dependent manner (Supplementary Fig. 2A, B). Application of erastin resulted in a significant increase in [Ca^2+^]_m_, which was significantly attenuated by application of both compounds. As expected, the application of erastin produced an increase in lipid peroxidation levels (Supplementary Fig. 2C, D). RR treatment alone caused no significant changes in mitochondrial ROS levels when compared with control and attenuated erastin-induced elevation in mitochondrial ROS levels in a concentration-dependent manner (Supplementary Fig. 2E). Furthermore, the highest concentration of RR caused a decrease in mitochondrial ROS levels comparable to that of co-treatment with erastin and ferrostatin (Supplementary Fig. 2F). We observed a loss of ΔΨ_m_ following erastin treatment, which was restored when cells were treated with either RR (Supplementary Fig. 2G), or MX (Supplementary Fig. 2H) in the presence of erastin. Notably, co-treatment with RR/erastin and MX/erastin restored ΔΨ_m_ to similar levels as the control and ferrostatin together with erastin (Supplementary Fig. 2I). We also tested the antiferroptotic potential of RR and MX against glutamate induced ferroptosis in our model (Supplementary Fig. 2J, K). In conclusion, using RR and MX in conditions of erastin-induced cell death significantly prevented ferroptosis hallmarks and reduced [Ca^2+^]_m_ uptake.

### Erastin-induced ferroptosis impairs mitochondrial function

**Fig. 3:**
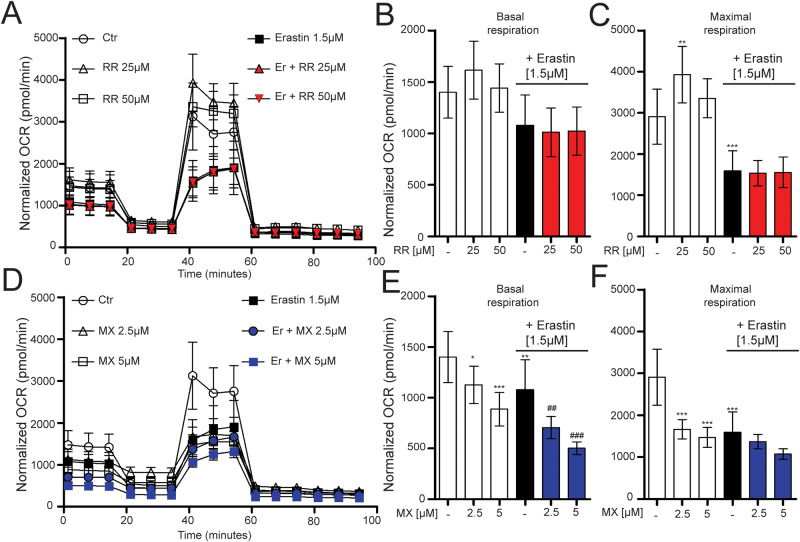
Ferroptosis induced by erastin and mitoxantrone impairs mitochondrial function. Measurement of the O_2_ consumption rate (OCR) in cells treated with RR (25 µM, 50 µM) (**A**–**C**), MX (2.5 µM, 5 µM) (**D**–**F**) in the presence or absence of erastin (1.5 µM), injected with the following compounds: oligomycin (4 µM), DNP (50 µM), rotenone (0.1 µM) and antimycin (1 µM), 2-DG (50 mM). *N* = 6–8 wells per condition. **B**, **C**, **E**, **F** analysis of OCR for basal respiration and maximal respiration. Data are presented as mean ± SD, Two-way ANOVA with Dunnett’s multiple comparisons test. *p < 0.05, **p < 0.01, ***p < 0.001 compared to control, ^##^*p* < 0.01, ^###^*p* < 0.001, compared to erastin alone.

Ferroptosis leads to a reduction in mitochondrial metabolism [13]. Here, we evaluated the effects of erastin and RR or MX on mitochondrial respiration by measuring the O_2_ consumption rate (OCR). RR (25 and 50 µM) did not affect basal OCR (Fig. 3A–C), whereas MX resulted in a decrease in basal OCR (Fig. 3D–F). Erastin caused a reduction in basal OCR, an effect that was potentiated upon co-treatment with MX (Fig. 3D–F), whereas RR did not affect basal respiration in erastin-treated cells (Fig. 3A–C). Uncoupling the mitochondrial respiratory chain from ATP synthesis by DNP, removes the limitation of proton re-entry, resulting in an increase in OCR, representative of maximal OXPHOS capacity. Erastin-treated cells exhibited a significant decrease in maximal OXPHOS compared with control. Interestingly, RR alone increased maximal respiration compared to control cells and MX decreased the maximal respiration (Fig. 3C, F). Co-treated cells showed no significant change in maximal respiration when compared to cells that were treated with erastin alone. Overall, it is evident that co-treatment with erastin and MX or RR does not fully prevent the decrease in mitochondrial respiration initiated by erastin challenge.

Mitochondrial fragmentation is a major hallmark of ferroptosis and associated cell death paradigms in neuronal cells. Studies show that erastin-treated HT22 cells exhibit a dramatic increase in the fragmentation of mitochondria, (morphology termed as category III–IV mitochondria), when compared to control cells, that mainly consist of elongated mitochondria (termed as categories I and II) [14]. Cells treated with erastin displayed mitochondria fragmentation and accumulation around the nucleus, regarded as category III and IV (Supplementary Fig. 3A, B). Mitochondrial morphology was preserved in cells co-treated with RR and erastin, resulting in the restoration of the mitochondrial morphology similar to control. In conclusion, erastin treatment led to fragmentation of mitochondria, RR preserved mitochondrial morphology in erastin-treated cells, suggesting a protective role in erastin-induced mitochondrial damage.

### Ruthenium-derived compound Ru265 is protective against ferroptosis

**Fig. 4:**
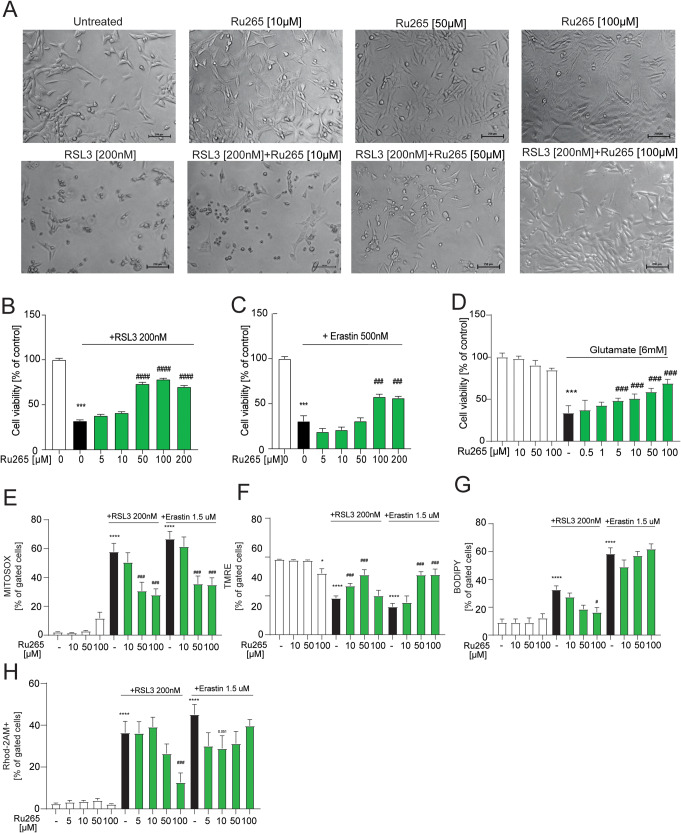
Antagonism of MCU using ruthenium-derived compound Ru265 is protective against ferroptosis. **A** Representative micrographs of three independent experiments with similar results of HT-22 cells treated with RSL3 and/or Ru265 at different concentrations. Scale bar, 250 μm. **B** MTT measurement showing cell viability after co-treatment with RSL3 and Ru265 increasing concentrations. **C** MTT measurement showing cell viability after co-treatment with erastin and Ru265 increasing concentrations. **D** MTT measurement showing cell viability after co-treatment with glutamate and Ru265 increasing concentrations. **E** MITOSOX staining upon treatment with Ru265 alone, and in co-treatment with RSL3 or erastin. **F** TMRE staining upon treatment with Ru265 alone, and in co-treatment with RSL3 or erastin. **G** BODIPY staining upon treatment with Ru265 alone, and in co-treatment with RSL3 or erastin. **H** Rhod-2AM staining upon treatment with Ru265 alone, and in co-treatment with RSL3 or erastin. Data are presented as mean ± SEM. *p < 0.05, **p < 0.01, ***p < 0.001, compared to control, ^###^*p* < 0.001, compared to ferroptotic stimuli (two-way ANOVA with Dunnett’s multiple comparisons test).

Since RR can act on various channels, such as the two-pore-domain K^+^ channels (TASK-3, TREK-2, TRAAK), TRPV4, ryanodine receptors, Piezo; and MX has effects on DNA synthesis, we investigated a cell permeable new diruthenium compound, Ru265 proposed to be more effective in the inhibition of the MCU than RR in permeabilized cells [35, 52]. Following Ru265 co-treatment with RSL3, cell morphology was preserved, comparable to untreated cells (Fig. 4A). Similarly, MTT assays demonstrated that Ru265 mediates a dose-dependent protection against the ferroptotic inducers RSL3, erastin and glutamate (Fig. 4B–D). Ru265 was able to prevent the hallmarks of ferroptosis in our model, namely mitochondrial superoxide (Fig. 4E), ΔΨ_m_ measurements (Fig. 4F) elicited by both erastin and RSL3. Lipid peroxidation measurements revealed that Ru265 reduced lipid peroxidation induced by RSL3 challenge, while in erastin-treated cells this protective effect was not detected (Fig. 4G). Flow cytometry analysis revealed a robust decrease of glutamate-induced PI incorporation (Supplementary Fig. 4A, B). In addition, Ru265 reduced the [Ca^2+^]_m_ uptake (Supplementary Fig. 4C, D) in the presence of glutamate. Ru265 also decreased the [Ca^2+^]_m_ uptake elicited by erastin and RSL3 both compounds (Fig. 4H and Supplementary Fig. 4E). Although in concentrations higher than 50μM, Ru265 led to a slight mitochondrial depolarization, it protected the cells in conditions of glutamate, erastin or RSL-3 stimuli. In HT-22 cells, the protective concentrations were higher than 10 μM, depending on the type of the stimulus, and we cannot exclude off-target effects due to these relatively high concentrations. All in all, Ru265 prevented ferroptosis in HT22 cells.

Additional experiments testing DS16570511, a small-molecule known to inhibit MCU [53], showed no effect in preventing erastin-induced ferroptosis (Supplementary Fig. 5A). This compound was shown to act on multiple auxiliary subunits within the complex [54]. However, the binding site of DS16570511 is not known yet. We then, measured [Ca^2+^]_m_ and observed that DS16570511 increases Rhod-2AM fluorescence in a concentration-dependent manner (Supplementary Fig. 5B, C).

### [Ca^+2^]_m_ uptake inhibition with the MICU1-targeting compound MCU-i4 is protective against ferroptosis in HT22 cells

**Fig. 5:**
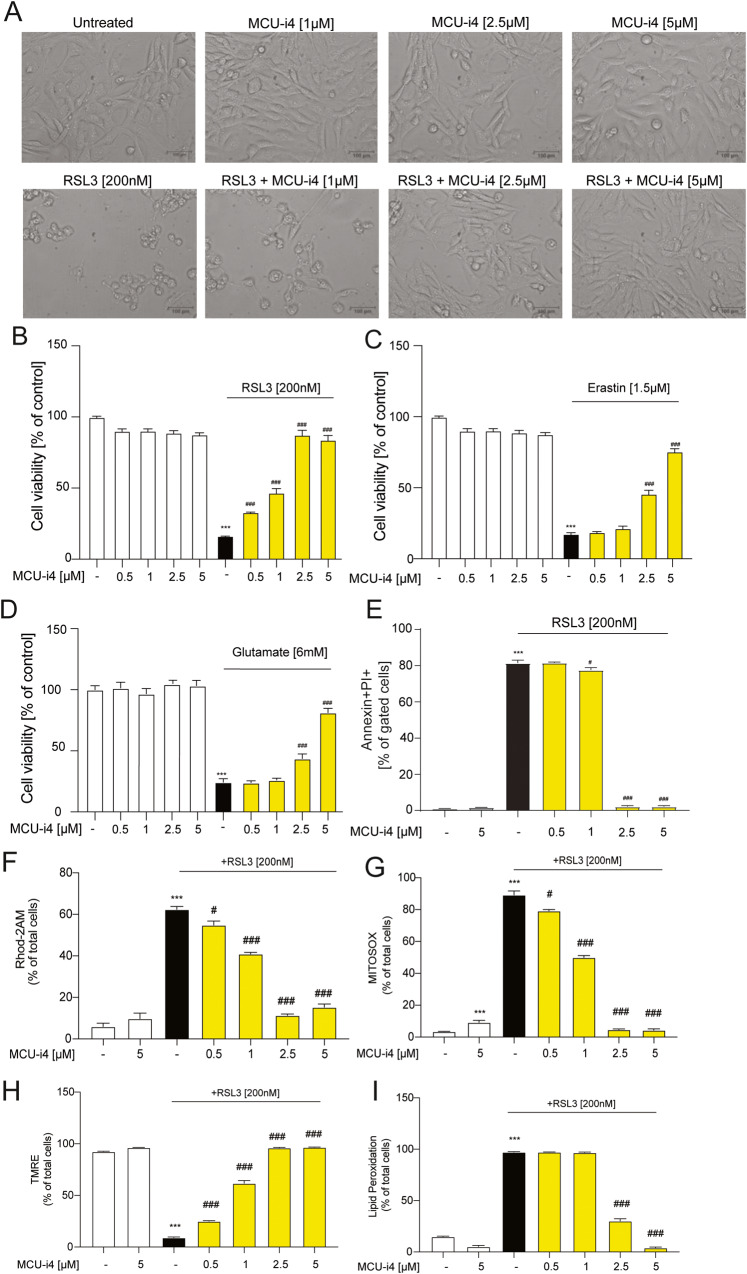
MICU1-targeting compound MCU-i4 protects HT22 cells against ferroptosis. **A** Microscope images of cells treated with MCUi4 and co-treated with RSL3 (200 nM). Scale bar = 100 µm. **B** MTT measurement showing cell viability after MCU-i4 (500 nM–5 µM) and RSL3 (200 nM) treatment. **C** MTT measurement showing cell viability after MCU-i4 (500 nM–5 µM) and erastin (1.5 µM) treatment. **D** MTT measurement showing cell viability after MCU-i4 (500 nM–5 µM) and glutamate (6 mM) treatment. **E** Quantification of Annexin/PI double positive cells upon treatment with MCU-i4 alone, and in co-treatment with RSL3. **F** Rhod-2AM staining upon treatment with MCU-i4 alone, and in co-treatment with RSL3. **G** MITOSOX staining upon treatment with MCU-i4 alone, and in co-treatment with RSL3. **H** TMRE staining upon treatment with MCU-I4 alone, and in co-treatment with RSL3. **I** BODIPY staining upon treatment with MCU-I4 alone, and in co-treatment with RSL3. Data is presented as mean ± SEM. ***p < 0.001, compared to control, ^#^*p* < 0.05, ^###^*p* < 0.001, compared to ferroptotic stimuli. (Two-way ANOVA with Dunnett’s multiple comparisons test).

Recently, MCU-i4 and MCU-i11 were detected in a high-throughput screening [55] of molecules that decreased [Ca^+2^]_m_ uptake. Unlike Ruthenium-containing compounds, or mitoxantrone, MCU-i4 and MCU-i11 target MICU1 and not the MCU channel per se. This unique targeting allowed us to interrogate modulation of mitochondrial calcium uptake through another molecular player, as Ruthenium-containing compounds can lack specificity at higher concentrations blocking other cation channels. We co-treated HT22 cells with several ferroptotic inducers RSL3 (Fig. 5A, B), erastin (Fig. 5C), and glutamate (Fig. 5D) and the MICU1 targeting compound MCU-i4 observing a dose-dependent effect in the prevention of cell death by MTT assays. When we tested MCU-i11, we observed that it was not able to prevent ferroptotic cell death (Supplementary Fig. 5D) and had no attenuating effects on [Ca^2+^]_m_ (Supplementary Fig. 5E, F). Furthermore, PI incorporation assays with flow cytometry demonstrated a decrease in PI positive cells with MCU-i4 in a concentration-dependent manner when cells were challenged with RSL3 (Fig. 5E).

### MCU-i4 prevents main ferroptotic hallmarks in HT22 cells

To follow up on the protective capacity of MCU-i4 to prevent ferroptotic cell death, we assessed the battery of ferroptosis hallmarks by flow cytometry. We assessed [Ca^+2^]_m_ uptake with Rhod-2AM and found an attenuation of Rhod-2AM fluorescence when the cells are exposed to RSL3 with increasing concentrations of MCU-i4 (Fig. 5F). Analysis of mitochondrial superoxide production demonstrated a robust concentration-dependent decrease upon RSL3 and MCUi4 co-treatment (Fig. 5G). Interestingly, treatment with MCU-i4 alone was enough to induce a mild increase on MitoSOX fluorescence, suggesting an increase in mitochondrial superoxide production (Fig. 5G). As in other cell systems, MCU-i4 was shown to depolarize mitochondrial potential and we tested whether this is the case as well in HT22 cells. Analysis of the TMRE fluorescence, showed that MCU-i4 alone did not change mitochondrial potential in HT22 cells. However, the loss of ΔΨ_m_ mediated by cell death stimuli was prevented with MCU-i4 similarly to the control levels (Fig. 5H). The increase in lipid peroxidation was strongly decreased upon MCU-i4 treatment, in a concentration dependent manner, starting at 2.5 μM, when RSL3 was present (Fig. 5I). These results demonstrate that MCU-i4 is able to revert main ferroptotic cellular hallmarks in conditions of ferroptosis.

### Ru265 and MCU-i4 prevent mitochondrial dysfunction in ferroptosis

**Fig. 6:**
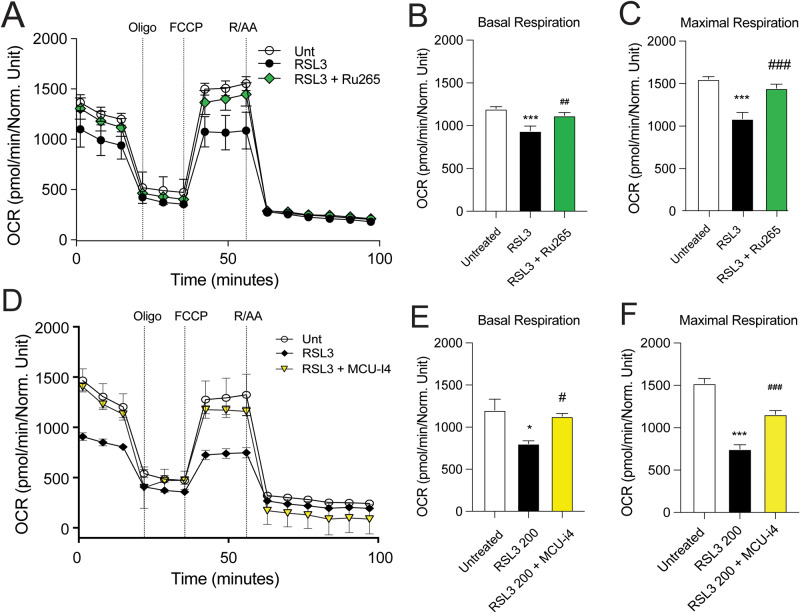
RSL3 impairs mitochondrial function and co-treatment with Ru265 and MCU-i4 restore it in HT-22 cells. Measurement of the O_2_ consumption rate (OCR) in cells treated with Ru265 (**A**–**C**), MCU-i4 (**D**–**F**) in the presence or absence of RSL3, injected with the following compounds: oligomycin (4 µM), DNP (50 µM), rotenone (0.1 µM) and antimycin (1 µM), 2-DG (50 mM). *N* = 6–8 wells per condition. **B**, **C**, **E**, **F** analysis of OCR for basal respiration and maximal respiration. Data are presented as mean ± SEM, *p < 0.05, ***p < 0.001 compared to control, ^#^*p* < 0.05, ^##^*p* < 0.01, ^###^*p* < 0.001, compared to RSL3. (Two-way ANOVA with Dunnett’s multiple comparisons test).

As RR and MX were not able to prevent the mitochondrial dysfunction phenotype in HT22 cells, we were interested in whether more specific compounds in modulating MCU complex activity could prevent mitochondrial dysfunction in ferroptosis. We observed that Ru265 was able to prevent the RSL3-induced deficits in respiration (Fig. 6A–C). Similarly, we observed a recovery in oxygen consumption ratio with MCU-i4 (Fig. 6D–F). Taken together, these data provide a comparison between the compounds that bind to the MCU complex (RR and MX) and the later developed Ru265 and MCU-i4 compounds. RR and MX can normalize ferroptosis hallmarks, but not the mitochondrial phenotype, while Ru265 and MCU-i4 can prevent ferroptotic cell detah and rescue the mitochondrial function.

### MICU1 is protective against ferroptosis in HT22 and MEF cells

**Fig. 7:**
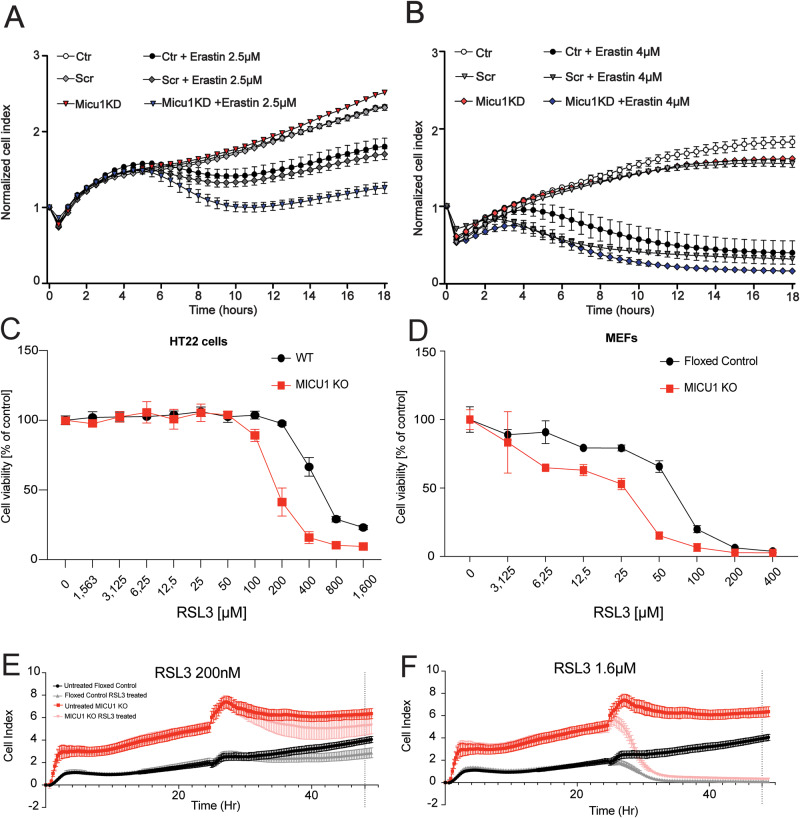
MICU1 is protective against ferroptosis. **A** xCELLigence real-time impedance measurements of cells transfected with either scrambled siRNA or MICU1 siRNA, treated with different concentrations of erastin such as (**A**) 1.5 µM and (**B**) 4 µM. **C** MTT assay of MICU1 KO HT22 and control cells treated with ascending concentrations of RSL3 (1.563 nM–1.6 µM), resulting in a dose-dependent decrease in cell viability. **D** MTT assay of MICU1 KO MEF cells and floxed control cells treated with ascending concentrations of RSL3 (3.125 nM–400 µM), resulting in a dose-dependent decrease in cell viability. xCELLigence real-time impedance measurements of MICU1 KO MEF cells and floxed controls grown for 24 h and then treated with different concentrations of RSL3 (200 nM (**E**)–1.6 µM (**F**) for 24 h. Data are presented as mean ± SEM.

Since small compounds may be subject to non-specific effects at high concentrations, we next investigated whether attenuated MCU complex expression could affect the cells’ sensitivity to ferroptosis. To this end, we used siRNA targeting MICU1. MICU1 functions as a key mediator of gatekeeping and independent activator of the channel, stimulating MCU activity, and MICU1 deficiency has been shown to lead to [Ca^+2^]_m_ overload [28, 56]. Using xCELLigence real-time impedance measurements, we showed that siRNA-mediated MICU1 deficiency caused a higher sensitivity to erastin concentrations compared to non-targeting siRNA or control cells (Fig. 7A, B). Similarly, we have generated MICU1 KO HT22 cells (Supplementary Fig. 6A) and tested for RSL3 sensitivity, evidencing that these HT22 cells exhibit a shift to the left compared to the wild-type controls (Fig. 7C), demonstrating increased sensitivity in the absence of MICU1, and supporting the protective role of MICU1 against ferroptosis. To strengthen our findings, we employed MICU1 KO MEF cells [55] (Supplementary Fig. 6B–E). We have demonstrated that these cells have a higher [Ca^+2^]_m_ in basal conditions (Supplementary Fig. 6D) and also in the presence of RSL3 treatment (Supplementary Fig. 6E). RSL3-sensitivity of MICU1 KO MEF cells was greater than their floxed controls (Fig. 7D). Interestingly, MICU1 KO MEFs exhibited an increased cell index compared to their floxed controls (Fig. 7E, F). Collectively, these data support the gatekeeper role of MICU1 against [Ca^+2^]_m_ overload upon ferroptosis in various models and provide new insights for the MCU/MICU1 involvement in ferroptosis [57].

### MCU-i4 is protective against ferroptosis-induced loss of neuronal network in human and murine neurons

**Fig. 8:**
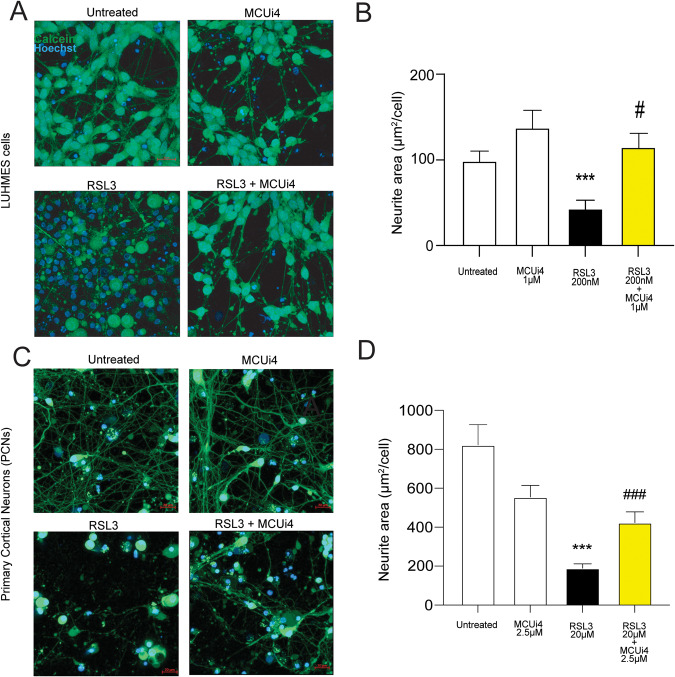
MCU-i4 prevents RSL3-induced neuronal network loss in dopaminergic and cortical neurons. **A** Confocal images of calcein and Hoechst staining on RSL3 (200 nM) and MCU-i4 (1 uM)-treated LUHMES cells. **B** Quantification of a maximal intensity projection of a Z-stack of live cells (*N* = 3 independent experiments; Untreated, *n* = 11 ROI; MCUi4, *n* = 11 ROI, RSL3, *n* = 11 ROI; RSL3, *n* = 11 ROI, RSL3 + MCUi4, *n* = 11 ROI). Data is presented as neurite area (µm^2^) per cell. Scale bar, 20 µm. Data presented are mean ± SEM. Statistical differences were detected using ANOVA with Dunnett’s T3 multiple comparisons test. **C** Confocal images of calcein and Hoechst staining on RSL3 (20 µM) and MCU-i4 (2.5 µM)-treated LUHMES cells. **D** Quantification of a maximal intensity projection of a Z-stack of live cells (*N* = 3 independent experiments; Untreated, *n* = 30 ROI; MCUi4, *n* = 20 ROI, RSL3, *n* = 30 ROI;, RSL3 + MCUi4, *n* = 30 ROI). Data are presented as neurite area (µm^2^) per cell. Scale bar, 20 µm. Data presented are mean ± SEM. Statistical differences were detected using ANOVA with Dunnett’s multiple comparisons test.

To test the effect of MCU-i4 in a human ferroptotic model system, we used the Lund Human mesencephalic (LUHMES) neuronal cells [58, 59]. LUHMES cells are human embryonic neuronal precursor cells that can be differentiated to postmitotic dopaminergic neurons, which allow for robust neuronal network analysis. We subjected LUHMES cells to RSL3 challenge and a co-treatment of RSL3 and MCUi4, measuring neurite degeneration by means of calcein fluorescence analysis. The neuronal networks of LUHMES cells exhibited important fragmentation upon RSL3 treatment, regarded as neuronal degeneration (Fig. 8A, B). Co-treatment of MCU-i4 prevented this RSL3-induced neuronal network degeneration (Fig. 8A, B), demonstrating that negative modulation of MCU complex could prevent dopaminergic cell death in ferroptotic conditions. To strengthen our findings, we used primary cortical neurons (PCNs) and subjected them to a similar analysis of neurite network. Our analysis yielded loss of neuronal network in PCNs upon RSL3 treatment and recovery of such phenotype with the co-treatment of MCU-i4 (Fig. 8C, D). Overall, these data demonstrate that MCU-i4 is protective across human and mouse in vitro models of ferroptosis.

## Discussion

In the present study, we demonstrated that using a panel of pharmacological compounds commonly used to target the MCU complex had protective effects in murine and human models of ferroptosis. Our data characterized hallmarks of ferroptosis, such as increases in [Ca^2+^]_m_, mitochondrial ROS, lipid peroxidation, ΔΨ_m_ and cell death upon ferroptosis induction. Erastin application also induced a morphological change in the mitochondria, resulting in mitochondrial fragmentation. The main ferroptotic hallmarks and decrease of viability were attenuated in the presence of these negative modulators of MCU complex. We demonstrated that MICU1 expression is protective against ferroptosis. Finally, we also demonstrated that MCU-i4, biding to MICU1, prevents ferroptosis in human and murine neurons.

RR could affect other ion channels and bind to other cellular sites such as ER, affecting both [Ca^2+^]_m_ and [Ca^2+^]_c_ [54, 60–62]. With the aid of novel HTS techniques, MX has been identified as a selective inhibitor of the MCU [33]. While both compounds rescued ferroptotic hallmarks such as [Ca^2+^]_m_, lipid peroxidation and membrane potential loss to similar degrees, MX reduced cell viability and metabolic rate when administered alone. MX effect on cell viability may be due to its function on DNA Topoisomerase II, as this drug was first introduced as an anticancer agent [63]. However, there are reports of neuroprotective potential of MX against AD-like pathology in primary murine neurons [64]. MX has been repurposed for multiple sclerosis, another neurodegenerative disease [65]. Here, we demonstrated that MX treatment decreased [Ca^2+^]_m_, but also mitochondrial respiration, which was accompanied by a significant disruption of ΔΨ_m_ and a small increase in early apoptotic cells. Previous studies have also observed ΔΨ_m_ disruption and reduced cell viability following treatment with MX in SH-SY5Y cells [66]. Furthermore, it has been proposed that the DNA Topoisomerase II-binding moiety and the MCU binding moiety are different [33]. Therefore, future rational drug design can be aimed at reducing the cytotoxicity of MX, while ensuring its MCU-binding activity.

MX and RR treatment could affect mitochondrial morphology and bioenergetics. Similar to previous studies [14], erastin treatment led to a substantial reduction in mitochondrial respiration. The OCR of RR-treated cells combined with erastin did not differ from erastin alone. We observed a reduction in OCR mediated by MX, comparable to levels present in erastin-treated mouse hippocampal cells, while previous studies have demonstrated that MX antagonism of the MCU in isolated liver mitochondria and HeLa cells caused no impairment of mitochondrial bioenergetics [33]. More recently, MX was employed in a mouse model of liver steatosis that was induced by high-fat diet, and it was able to fully prevent decreases of the OCR in vitro in HepG2 cells treated with oleic acid and palmitic acids [67]. Additional investigations are necessary, using more representative cellular models for brain pathologies such as primary cultured neurons and induced pluripotent stem cell-derived brain cells, or in vivo models for neurodegeneration to test the antiferroptotic effects of MX.

The novel ruthenium-derived compound Ru265 was generated by Woods et al. [52], where structural modifications were introduced to obtain more potent MCU inhibitors. Ru265 modifications increase the compound’s cell permeability, and specificity for MCU without affecting cytosolic calcium. Mechanistic studies demonstrated that a cysteine mutation in the N-terminal matrix motif of MCU (C97A) suppresses the MCU inhibitory effect of Ru265. Márta et al. tested Ru265 in intact HeLa cells and reported limited potential to attenuate histamine-induced [Ca^2+^]_m_ increases [68], which could be attributed to the high inter-lab variability of HeLa mutations [69], therefore we can not exclude off-target effects independent of calcium signaling. In our studies, Ru265 effectively reverted the glutamate-induced cell death phenotype starting at 5 μM, and at higher concentrations in the presence of RSL-3 or erastin in murine HT22 cells. The oxygen-glucose deprivation (OGD) model, an in vitro model of [Ca^2+^]_m_ overload, used by Novorolsky et al. [35] demonstrated protective properties of Ru265 against lethal OGD in primary cortical neurons. However, in vivo experiments using the hypoxia ischemia model, showed a dose-dependent increase in seizure-like behaviors at higher concentrations, indicating that Ru265 may have effects on calcium buffering upon chronic administration. Our data and the study of Novorolsky et al. support the further investigation of Ru265 for therapeutic purposes, such as site-specific delivery that could widen its therapeutic window [35].

The study of Di Marco et al. investigated short-term calcium fluxes within a timeframe of minutes and a 24 h treatment in ex vivo muscle fiber growth. Until this study, MCU-i4- or MCU-i11-mediated [Ca^2+^]_m_ attenuation has not been assessed in neurons or in ferroptosis in vitro models, where [Ca^2+^]_m_ increases occur within the first 4–6 h and elicit an effect on viability after 16–18 h. Our study represents the first study that employs the MICU1 binding compound MCU-i4 in mouse and human neurons. Interestingly, MCU-i4 alone has no detrimental effect in neurite area in human dopaminergic neurons. These findings suggest that different cell types that rely on calcium fluxes react differentially to [Ca^2+^]_m_ in physiological (muscle fiber growth) [55, 70] and pathological conditions (ferroptotic cell death) (this study). An interesting piece of data: MCU-i4 alone mildly increases mitochondrial ROS generation. This finding implies that there is a potential mitohormetic effect of MCU-i4 downstream of [Ca^2+^]_m_ uptake inhibition. This potential mechanism remains to be investigated.

While MCU inhibition is protective against erastin, using siRNA specific to MICU1 genes, we showed that MICU1 knockdown cells exhibited more sensitivity toward erastin treatment. Parallelly, MICU1 KO MEFs and HT22 cells exhibit increased sensitivity to ferroptosis. This is in agreement with studies showing that MICU1 deletion sensitizes human cells to manganese-dependent cell death by disinhibiting MCU-mediated manganese uptake, identifying a critical contribution of MICU1 to the uniporter selectivity [71–73]. The human and murine MICU1 proteins are 1:1 orthologs and exhibit a high degree of homology, with a 93% match between their amino acid sequences, with minor differences of residues [74] (Supplementary File 1).

MICU1 forms a homodimer with itself and a heterodimer with MICU2 and MICU3, while MICU2 and MICU3 cannot form a heterodimer. Recently, Singh et al. [75] showed that neuron-specific MICU1-deficient mice exhibit neurodegeneration and progressive cognitive decline, with MICU1-deficient patient-derived cells exhibit dysregulated calcium signaling and increased cell death. Our findings are contributing to the understanding of the mechanisms that allow MICU1 to support cell survival by preventing [Ca^2+^]_m_ overload and contributing to cell demise in MICU1 deficiency.

MICU1 is usually regarded as a gatekeeper of [Ca^2+^]_m_ overload in basal conditions. However, its calcium sensing functions promote [Ca^2+^]_m_ uptake upon increases of [Ca^2+^]_c_ [76]. Taking these findings into account, we can compare our cytoprotective findings of MICU1 in small-molecule-induced ferroptosis, with the study of Nakamura et al. [77] demonstrating a cytoprotective role of MICU1 in cold-induced ferroptosis in the cancer cell line A549. These findings indicate that MICU1 may have cell-state-specific implications in ferroptosis, as ferroptosis plays roles in a variety of pathologies, including neurodegeneration and cancer.

## Concluding remarks

In conclusion, the present study illustrates that mitochondrial damage is essential for the cell death pathway of ferroptosis in neuronal cells. Our results demonstrate that targeting mitochondria by the negative modulation of MCU complex is protective against ferroptosis. RR and MX findings should be interpreted with caution as they may have off-target effects. Ru265 and MCU-i4 are effective in promoting neuroprotection against ferroptosis. Although the basal mitochondrial calcium level was not affected by Ru265, or MCU-i4 alone, while RR and MX decreased the basal mitochondrial calcium levels, all these compounds reduced mitochondrial calcium overload mediated by ferroptotic stimuli. MICU1-deficient in vitro models demonstrate a role for MICU1 involvement in ferroptosis. In conclusion, this study provides the foundation for further investigation into the therapeutic potential of the negative modulation of MCU complex against ferroptosis, by setting the foundation of screening extensive libraries of small molecules targeting MCU complex.

### Supplementary information

Supplementary figures

Supplemental file

Western blot uncropped

Checklist CDD

## Data Availability

The datasets generated during and/or analyzed during the current study are available from the corresponding author on reasonable request.
