# Supplementary material for: Negative modulation of mitochondrial calcium uniporter complex protects neurons against ferroptosis
Source: Cell Death Dis. 2023 Nov 25;14(11):772. doi: 10.1038/s41419-023-06290-1 (PMC10676387; doi:10.1038/s41419-023-06290-1)
Supplement: Supplementary file 1 — Supplementary figures [file 41419_2023_6290_MOESM1_ESM.docx]

**SUPPLEMENTARY FIGURES**

# NEGATIVE MODULATION OF MITOCHONDRIAL CALCIUM UNIPORTER COMPLEX PROTECTS NEURONS AGAINST FERROPTOSIS

Alejandro Marmolejo-Garza^1,2†^, Inge E. Krabbendam^1†^, Minh Danh Anh Luu^1^, Famke Brouwer^1^, Marina Trombetta-Lima^1,2^, Osman Unal^1^, Shane J. O’Connor^1^, Naďa Majerníková^1,3^, Carolina R.S. Elzinga^1^, Cristina Mammucari^4^, Martina Schmidt^1^, Muniswamy Madesh^5^, Erik Boddeke^2^, and Amalia M. Dolga^1^*

Author affiliations:

^1^Faculty of Science and Engineering, Department of Molecular Pharmacology, Groningen Research Institute of Pharmacy (GRIP), University of Groningen, 9713 AV Groningen, The Netherlands

^2^Department of Biomedical Sciences of Cells & Systems, section Molecular Neurobiology, Faculty of Medical Sciences, University of Groningen, University Medical Center Groningen, Groningen, The Netherlands

^3^Department of Pathology and Medical Biology, University Medical Centre Groningen, University of Groningen, Groningen, The Netherlands

^4^Department of Biomedical Sciences, University of Padua, 35131 Padua, Italy

^5^Department of Medicine/Cardiology, Center for Mitochondrial Medicine, University of Texas Health San Antonio, San Antonio, Texas 78229, United States

^†^These authors contributed equally

***Corresponding author**: A. M. Dolga, Faculty of Science and Engineering, Groningen Research Institute of Pharmacy, Department of Molecular Pharmacology, University of Groningen, Antonius Deusinglaan 1, Groningen, The Netherlands.

A. M. Dolga Tel: +31 50363 6372; Fax: +31 50 363 6908; E-mail: a.m.dolga@rug.nl,

**Supplementary Figure 1. HT22 cells undergo ferroptotic cell death upon treatment with Erastin, RSL3 or Glutamate.**

A. Representative micrographs three different experiments with similar results of HT-22 cells treated with Erastin, RSL3 and Glutamate. B. MTT measurements showing cell viability after treatment with Erastin. C. MTT measurements showing cell viability after treatment with the ferroptosis inhibitor PD-146178 in the presence or absence of Erastin. D. MTT measurements showing cell viability after treatment with the ferroptosis inhibitor ferrostatin in the presence or absence of Erastin. E. MTT measurements showing cell viability after Glutamate treatment in the presence or absence of the ferroptosis inhibitor PD-146178. F. MTT measurements showing cell viability after Glutamate treatment in the presence or absence of the ferroptosis inhibitor ferrostatin. G. MTT measurements showing cell viability after RSL3 treatment in the presence or absence of the ferroptosis inhibitors PD-146178 or ferrostatin. N=6. For B, one-way ANOVA was performed. For C-G, two-way ANOVA with Dunnett’s multiple comparison test were performed. Data are presented as mean±SD ^***^p<0.001, compared to untreated control, ^###^p<0.001, compared to ferroptotic inducer alone.

**Supplementary Figure 2. Supplemental data on antiferroptotic effects of RR and MX in HT22 cells.**

A-B. Rhod-2 fluorescence, a readout for [Ca^2+^]_m,_ after treatment with RR (25-100µM) (A) or MX (2.5-10µM) (B) with or without erastin (1.5µM). C-D. Lipid peroxidation measurements (BODIPY) after treatment with RR (C) or MX (D) with or without erastin.  E-F. Treatment with RR (10-25µM) (E) or Fer (5µM) with or without erastin (F) following MitoSOX staining for mitochondrial ROS production. G-I. TMRE measurements indicating membrane depolarization after treatment with RR (10-25µM) (G), MX (2.5-5µM) (H) or Fer (5µM) (I) with or without erastin. J. MTT measurement of Ruthenium Red (RR) in the presence or absence of Glutamate. K. MTT measurement of Mitoxantrone (MX) in the presence or absence of Glutamate.N=6. Two-way ANOVA with Dunnett’s multiple comparison test were performed. Data are presented as mean±SD *p<0.05, **p<0.01, ***p<0.001, compared to control, ^###^p<0.001, compared to erastin alone control.

**Supplementary Figure 3. Erastin-induced ferroptosis disrupts mitochondrial morphology and RR is capable of preventing it.**

A-B. Mitochondrial morphology with (A) examples of each treatment condition: control, erastin (1.5µM), RR (25µM), RR + erastin. 40x magnification. B. Stacked bar graph showing the ratio of each category (I-IV) in each condition.

**Supplementary Figure 4.** **Additional data on antagonism of MCU using rutenium-derived compound Ru265 is protective against ferroptosis.**

A. Representative scatterplots depicting PE and FITC fluorescence of three independent experiments with similar results of HT-22 cells treated with Glutamate and/or Ru265 and stained with Annexin V and PI. B. Quantification of AnnexinV+/PI+ cells HT-22 cells treated with Glutamate and/or Ru265. C. Representative histograms depicting Rhod-2AM fluorescence on HT-22 cells treated with glutamate and/or Ru265. D. Quantification of Rhod-2AM positive cells from (C) represented as a % of cells E. Rhod-2AM quantification of Figure 4H represented as mean fluorescence intensity (MFI). N=6-9 replicates. Two-way ANOVA with Dunnett’s multiple comparison test were performed. Data are presented as mean±SEM. ***p<0.001, compared to control, ^###^p<0.001, compared to ferroptotic stimuli.

**

**Supplementary Figure 5. Anti-ferroptotic activity of reported mitochondrial calcium modulators in HT-22 cells.**

A. MTT measurement of DS16570511 in the presence of Erastin. B. Rhod-2AM staining upon treatment with increasing concentrations of DS16570511. C. Rhod-2AM quantification of (D) represented as mean fluorescence intensity (MFI). D. MTT measurement of MCU-i11 in the presence or absence of RSL3. E. Rhod-2AM staining upon treatment with increasing concentrations of MCU-i11. F. Rhod-2AM quantification of (E) represented as mean fluorescence intensity (MFI). For B,C,E,F one-way ANOVA was performed. For A and D, two-way ANOVA with Dunnett’s multiple comparison test were performed. Data are presented as mean±SEM. ***p<0.001, compared to control, ^###^p<0.001, compared to ferroptotic stimuli.

**

**Supplementary Figure 6. Supplemental data on MICU1 deficiency**

A. Immunoblot depicting MICU1 loss of expression in clone 3 of HT22 cells. B. Immunoblot depicting MICU1 loss of expression in KO MEFs. C. qPCR analysis on MICU1 gene expression on MICU1 KO MEF cells. D. Rhod-2AM measurements on untreated MICU1 KO MEF cells compared to their floxed controls expressed in MFI E. MTT measurement showing cell viability after RSL3 (200nM) and classical ferroptosis inhibitors in MEF cells. F. Representative pictures of Rhod-2AM staining and Mitotracker green demonstrating co-localization of green and red signal. For panel C, data are presented as mean±SD, for panels C-D, data are presented as mean±SEM. For D, t-test was performed. For E, Two-way ANOVA with Dunnett’s multiple comparison test were performed. *p<0.001, ***p<0.001 compared to untreated floxed control ^###^p<0.001 compared to the ferroptotic stimuli within genotype. ^$$$^p<0.001 against floxed control in the same condition.
