# Supplementary material for: Negative modulation of mitochondrial calcium uniporter complex protects neurons against ferroptosis
Source: Cell Death Dis. 2023 Nov 25;14(11):772. doi: 10.1038/s41419-023-06290-1 (PMC10676387; doi:10.1038/s41419-023-06290-1)
Supplement: Supplementary file 2 — Supplemental file [file 41419_2023_6290_MOESM2_ESM.docx]

**Supplementary File 1. Homology between human and mouse MICU1 aminoacid sequences**

Filename: ZPP5PPTT11R-Alignment.txt

BLASTP 2.13.0+

Reference: Stephen F. Altschul, Thomas L. Madden, Alejandro

A. Schaffer, Jinghui Zhang, Zheng Zhang, Webb Miller, and

David J. Lipman (1997), "Gapped BLAST and PSI-BLAST: a new

generation of protein database search programs", Nucleic

Acids Res. 25:3389-3402.

Reference for compositional score matrix adjustment: Stephen

F. Altschul, John C. Wootton, E. Michael Gertz, Richa

Agarwala, Aleksandr Morgulis, Alejandro A. Schaffer, and

Yi-Kuo Yu (2005) "Protein database searches using

compositionally adjusted substitution matrices", FEBS J.

272:5101-5109.

RID: ZPP5PPTT11R

Query= NP_001182447.1 calcium uptake protein 1, mitochondrial isoform 2 [Homo

sapiens]

Length=476

Score E Max

Sequences producing significant alignments: (Bits) Value Ident

NP_659071.1 calcium uptake protein 1, mitochondrial isoform 3 ... 926 0.0 93%

ALIGNMENTS

>NP_659071.1 calcium uptake protein 1, mitochondrial isoform 3 [Mus musculus]

Length=477

Score = 926 bits (2394), Expect = 0.0, Method: Compositional matrix adjust.

Identities = 442/477 (93%), Positives = 460/477 (96%), Gaps = 2/477 (0%)

Query 1 MFRLNSLSALAELAVGSRWYHGGSQPIQIRRRLMMVAFLGASAVTASTGLLWKRAHAESP 60

MFRLN+LSALAELAVGSRWYHG SQP Q +RRLM+VAFLGASAVTASTGLLWK+AHAESP

Sbjct 1 MFRLNTLSALAELAVGSRWYHGASQPTQTKRRLMLVAFLGASAVTASTGLLWKKAHAESP 60

Query 61 PCVDNLKSDIGDKGKNKDEGDVCNHEKKTADLA--PHPEEKKKKRSGFRDRKVMEYENRI 118

PCV++ K D DK +NKD G+V + E + AD A P+PE+KKKKRSGFRDRKVMEYENRI

Sbjct 61 PCVNSKKPDTEDKERNKDSGEVSSREGRAADAAAEPYPEDKKKKRSGFRDRKVMEYENRI 120

Query 119 RAYSTPDKIFRYFATLKVISEPGEAEVFMTPEDFVRSITPNEKQPEHLGLDQYIIKRFDG 178

RAYSTPDKIFRYFATLKVI+EPGE EVFMTP+DFVRSITPNEKQPEHLGLDQYIIKRFDG

Sbjct 121 RAYSTPDKIFRYFATLKVINEPGETEVFMTPQDFVRSITPNEKQPEHLGLDQYIIKRFDG 180

Query 179 KKISQEREKFADEGSIFYTLGECGLISFSDYIFLTTVLSTPQRNFEIAFKMFDLNGDGEV 238

KKI+QEREKFADEGSIFY+LGECGLISFSDYIFLTTVLSTPQRNFEIAFKMFDLNGDGEV

Sbjct 181 KKIAQEREKFADEGSIFYSLGECGLISFSDYIFLTTVLSTPQRNFEIAFKMFDLNGDGEV 240

Query 239 DMEEFEQVQSIIRSQTSMGMRHRDRPTTGNTLKSGLCSALTTYFFGADLKGKLTIKNFLE 298

DMEEFEQVQSIIRSQTSMGMRHRDRPTTGNTLKSGLCSALTTYFFGADLKGKLTIKNFLE

Sbjct 241 DMEEFEQVQSIIRSQTSMGMRHRDRPTTGNTLKSGLCSALTTYFFGADLKGKLTIKNFLE 300

Query 299 FQRKLQHDVLKLEFERHDPVDGRITERQFGGMLLAYSGVQSKKLTAMQRQLKKHFKEGKG 358

FQRKLQHDVLKLEFERHDPVDGRI+ERQFGGMLLAYSGVQSKKLTAMQRQLKKHFK+GKG

Sbjct 301 FQRKLQHDVLKLEFERHDPVDGRISERQFGGMLLAYSGVQSKKLTAMQRQLKKHFKDGKG 360

Query 359 LTFQEVENFFTFLKNINDVDTALSFYHMAGASLDKVTMQQVARTVAKVELSDHVCDVVFA 418

LTFQEVENFFTFLKNINDVDTALSFYHMAGASLDKVTMQQVARTVAKVELSDHVCDVVFA

Sbjct 361 LTFQEVENFFTFLKNINDVDTALSFYHMAGASLDKVTMQQVARTVAKVELSDHVCDVVFA 420

Query 419 LFDCDGNGELSNKEFVSIMKQRLMRGLEKPKDMGFTRLMQAMWKCAQETAWDFALPK 475

LFDCDGNGELSNKEFVSIMKQRLMRGLEKPKDMGFTRLMQAMWKCAQETAWDFALPK

Sbjct 421 LFDCDGNGELSNKEFVSIMKQRLMRGLEKPKDMGFTRLMQAMWKCAQETAWDFALPK 477
