# Supplementary material for: Negative modulation of mitochondrial calcium uniporter complex protects neurons against ferroptosis
Source: Cell Death Dis. 2023 Nov 25;14(11):772. doi: 10.1038/s41419-023-06290-1 (PMC10676387; doi:10.1038/s41419-023-06290-1)
Supplement: Supplementary file 3 — Western blot uncropped [file 41419_2023_6290_MOESM3_ESM.pptx]

## Slide 1
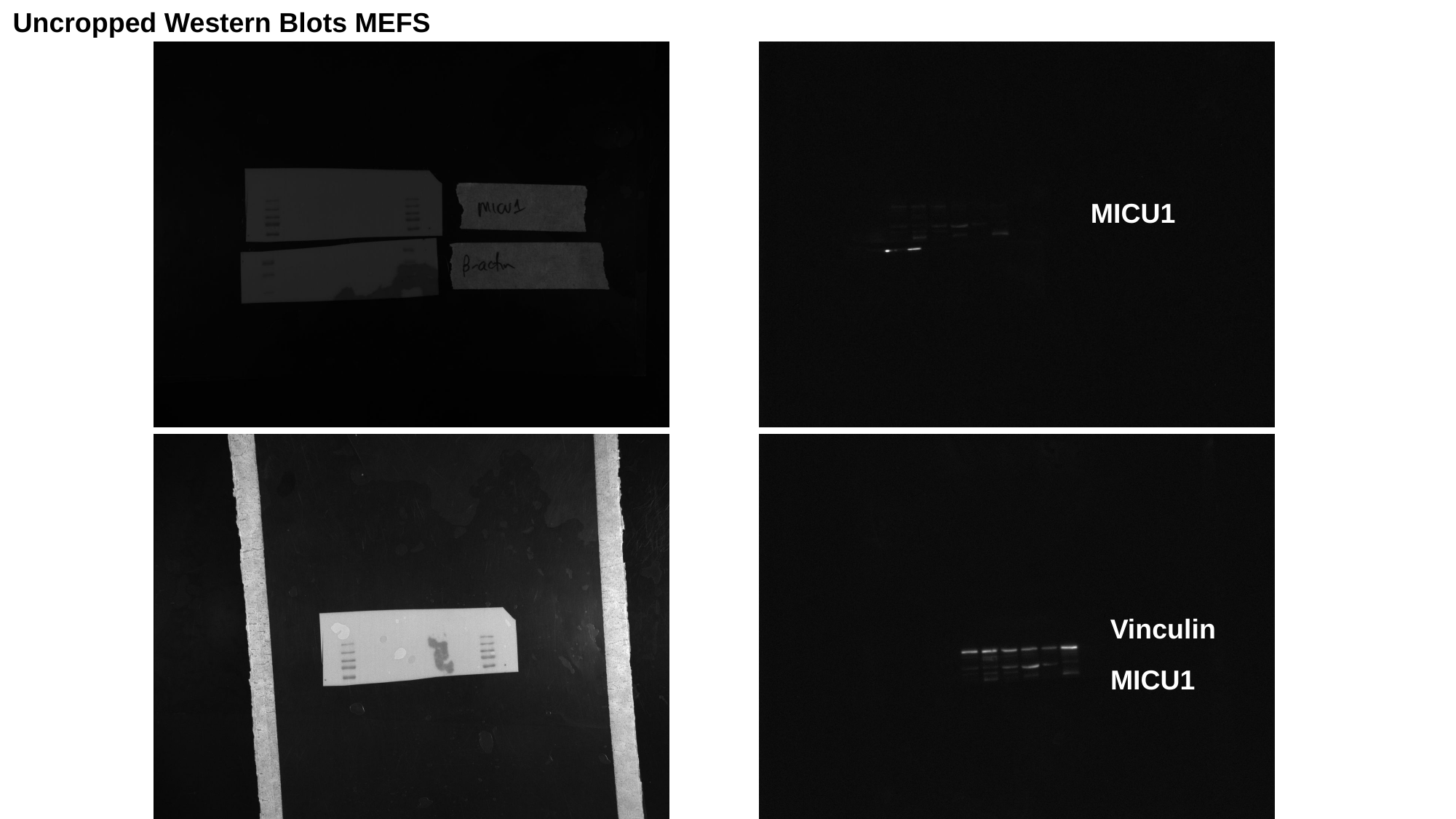

Uncropped Western Blots MEFS
MICU1
Vinculin
MICU1

## Slide 2
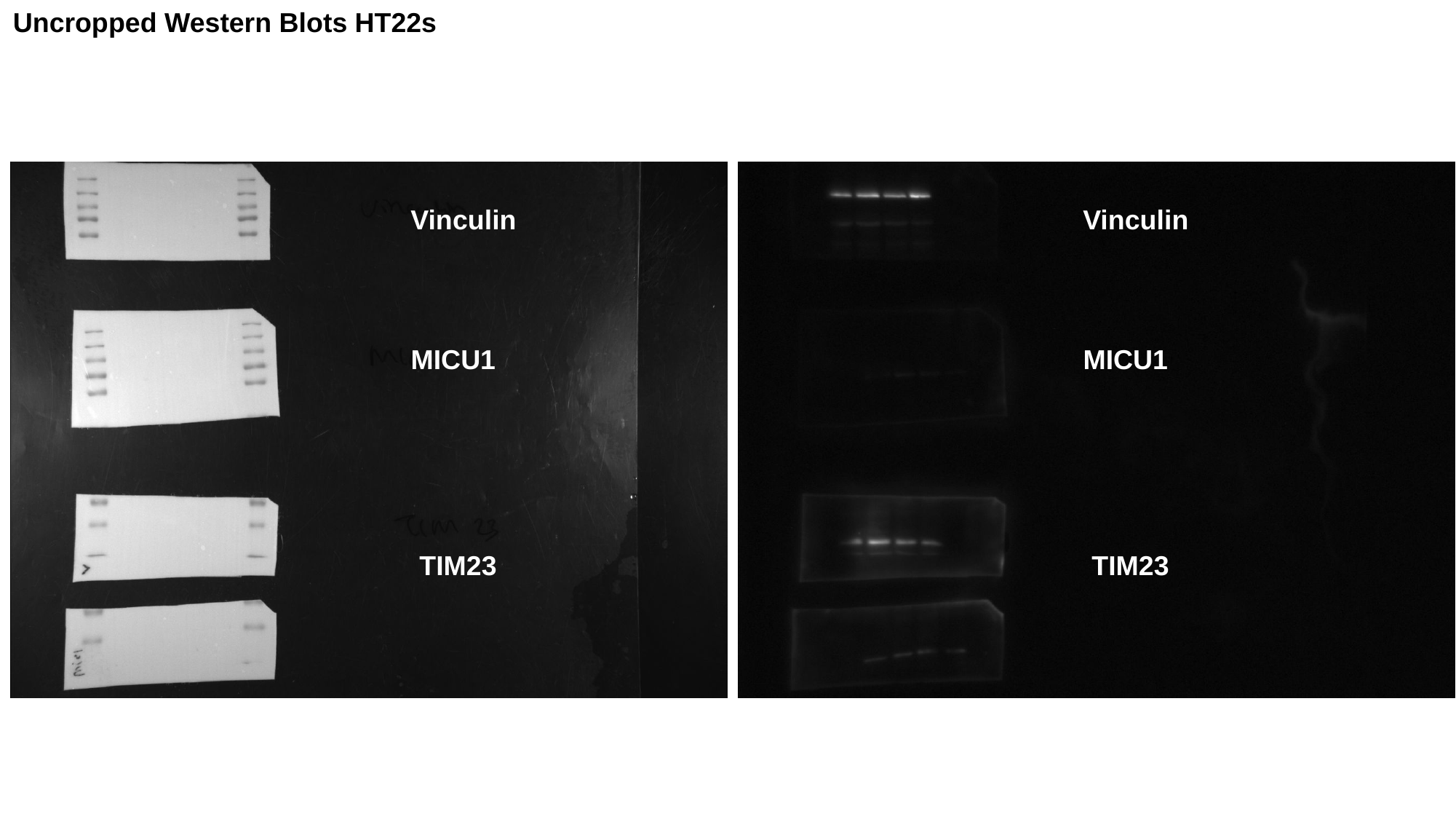

Uncropped Western Blots HT22s
Vinculin
Vinculin
MICU1
MICU1
TIM23
TIM23
